# Genetic and phylogenetic relationships analysis of the complete chloroplast genome *Cucumis sativus* to China

**DOI:** 10.1080/23802359.2019.1687040

**Published:** 2019-11-08

**Authors:** Wei Xiao, Xuanyao Wang, Mei Bie

**Affiliations:** Institute of Education, Changchun Normal University, Changchun, Jilin, PR China

**Keywords:** *Cucumis sativus*, Cucurbitaceae, chloroplast genome, genetic analysis, phylogenetic relationships

## Abstract

Cucumber, *Cucumis sativus* is one of the most cultivated vegetables in the world, which is mostly consumed raw in China. The complete chloroplast genome of *C. sativus* was assembled and annotated in this study. Its size was 155,525 bp, containing a large single-copy region of 86,878 bp, a small single-copy region of 18,269 bp and a pair of IR regions of 25,189 bp. Whole chloroplast genome of *C. sativus* contains 131 genes, including 77 protein-coding genes (PCGs), 46 transfer RNA genes (tRNAs), and 8 ribosome RNA genes (rRNAs). The overall nucleotide composition is: A of 31.1%, T of 31.9%, C of 18.8% and G of 18.2%, with a total GC content of the chloroplast genome 37.0% and AT of 63.0%. Genetic and phylogenetic analysis based on 10 plants species confirmed the position of *C. sativus* closely related to Cucurbitaceae species of *Cucumis hystrix*.

Cucumber, *Cucumis sativus* is a widely cultivated plant in the gourd family, Cucurbitaceae, which is a creeping vine that bears cucumiform fruits that are used as vegetables. *Cucumis sativus* is not only an economically important cultivated plant, but also a model system for studies on sex determination and plant vascular biology (Tanurdzic and Banks 2004; Liu et al. 2008). Cucumber was originated from South Asia, but now it is cultivated in most of the continents. The Cucumber was cultivated in Western Asia for at least 3000 years and then introduced by Romans to other parts of Europe (Garcia-Mas et al. 2012). Cucumber is rich in water content which helps to hydrate the body. It is rich in nutrient content which prevents from various health problems such as skin health, prevent kidney stones and constipation, maintains blood pressure, and prevents diabetes and body function (Staub et al. 1999). So, in this review, we assembled and annotated the complete chloroplast genome of *C. sativus* and discussed genetic and phylogenetic relationship with other plants, which provides a valuable resource for developing elite cultivars and for studying the evolution and function of the plant vascular system.

The sample of *C. sativus* was collected in Cucumber picking garden in Changchun district of Jilin province (Changchun, Jilin, China, 125.41E; 43.92N). The fresh plant tissue and chloroplast (cp) DNA of *C. sativus* was extracted using the modified CTAB method and stored at Institute of Education, Changchun Normal University (No.IE-CNU-01). The cp DNA was purified and fragmented using the NEB Next Ultra^TM^ II DNA Library Prep Kit (NEB, BJ, CN), which was sequenced using the Illumina HiSeq X-ten Sequencing Platform (Illumina, San Diego, CA). Quality and adapters control was performed and removed low-quality reads and adapters using the NGS QC Toolkit software (Patel and Jain 2012). The chloroplast genome of *C. sativus* was assembled and annotated using the MitoZ software (Meng et al. 2019). The physical map of *C. sativus* chloroplast genome was generated using the OGDRAW version 1.3.1 (Greiner et al. 2019).

The complete chloroplast genome of *C. sativus* (GenBank accession no. MK8659751) was a circle with 155,525 bp in size, containing a large single-copy region (LSC) of 86,878 bp, a small single-copy region (SSC) of 18,269 bp, and a pair of inverted repeat regions (IRs) of 25,189 bp in each one. The cp DNA of *C. sativus* comprised 131 genes, including 77 protein-coding genes (PCGs), 46 transfer RNA genes (tRNAs) and 8 ribosomal RNA genes (rRNAs). Twenty-one genes were found duplicated in the one of the IR regions, including 8 PCGs (*rpl2*, *rpl23*, *ycf2*, *ycf15*, *ndhB*, *rps7*, *rps12* and *ycf1*), 9 tRNA genes (*trnI-CAU*, *trnL-CAA*, *trnV-GAC,* two of *trnI-GAU,* two of *trnA-UGC, trnR-ACG* and *trnN-GUU*) and 4 rRNA genes (*rrn16, rrn23, rrn4.5* and *rrn5*). The overall nucleotide composition is: 31.1% of A, 31.9% of T, 18.8% of C, and 18.2% of G, with a total GC content of 37.0% and AT of 63.0%.

The maximum-likelihood (ML) methods were used for genetic analysis and phylogenetic relationship of 10 plant species. The phylogenetic tree was reconstructed using RaxML version 8.0 (Stamatakis 2014) with the GTR + G + I model. Branch support was inferred using 2,000 bootstrap replicates. Phylogenetic relationship obtained with the ML approach were identical to those obtained using the Bayesian analysis. The Bayesina phylogenetic alignment was analyzed by MrBayes version 3.2.5 (Ronquist and Huelsenbeck 2003) based on the most appropriate model. The phylogenetic tree was performed using MEGA X software (Kumar et al. 2018) and edited using iTOL version 4.0 (https://itol.embl.de/) (Letunic and Bork 2019). As shown in the result of the phylogenetic ML tree (Figure 1), the chloroplast genome of *C. sativus* is clustered and closest to Cucurbitaceae species of *Cucumis hystrix* (KF957866.1) in the genetic evolutionary relationship.

**Figure 1.**
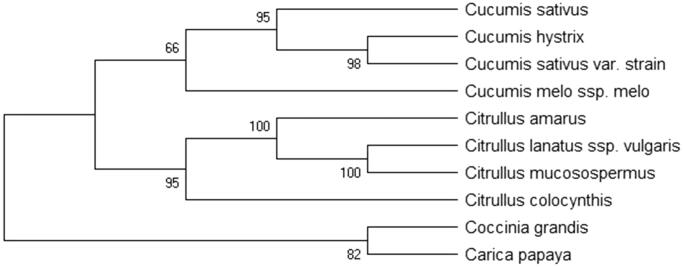
The maximum-likelihood (ML) phylogenetic tree from 10 species plants chloroplast genome. The length of branch represents the divergence distance. All the herbal medicine species chloroplast genomes in this study have been deposited in the GenBank and accession numbers are as follows: *Cucumis hystrix KF957866.1*, *Cucumis sativus var. strain KX231328.1*, *Cucumis melo ssp. melo JF412791.1*, *Citrullus lanatus ssp. vulgaris KY014105.1*, *Citrullus mucosospermus KY430686.1*, *Citrullus amarus MF536694.1*, *Citrullus colocynthis MF357889.1*, *Coccinia grandis KX147312.1*, *Carica papaya EU431223.1*.
